# Why Motive Matters: The Appraisal of Criminal Aims

**DOI:** 10.3390/bs15091244

**Published:** 2025-09-12

**Authors:** Keelah E. G. Williams, Ashley M. Votruba, Ross S. Eagle

**Affiliations:** 1 Department of Psychology, Hamilton College, Clinton, NY 13323, USA; 2 Department of Psychology, University of Nebraska—Lincoln, Lincoln, NE 68583, USA; ashley.votruba@unl.edu

**Keywords:** motive, criminal law, psychology, evolutionary psychology

## Abstract

In a strict legal sense, motive is often irrelevant in U.S. criminal law. Whether one smothered their grandmother with a pillow to ease her pain or to fraudulently collect her social security benefits, they are legally guilty of murder all the same. Yet anyone who has watched a courtroom drama or sat in the jury box knows the prominent role that establishing motive seems to play in influencing legal decision-makers. Why is motive so pivotal, so psychologically powerful for most people? We briefly review the existing literature on the psychology of motive, then introduce an adaptationist framework as a new lens for examining this question. In particular, we consider how motive assists perceivers in inferring actors’ welfare trade-off ratios, with important implications for legal judgments and willingness to punish.

## 1. Introduction

One does not need to look too far to evidence people’s preoccupation with criminal motive. Whether it is the news headline for the most recent mass shooting or the latest crime drama television series, our strong drive to understand why someone committed a heinous act is apparent. Based on this alone, one might think that motive is a critical element for proving guilt. In actuality, the role of motive in establishing criminal liability is exceedingly narrow, and often wholly irrelevant in American courtrooms (Hessick, 2006). Instead, in most cases, establishing criminal liability involves determining if the defendant committed the required actions for the offense (actus reus) with the prerequisite intent (mens rea; LaFave & Ohlin, 2023). Motive can be brought up during trial, but it has limited evidentiary value. It can be helpful for establishing the defendant committed an act, but it is not necessary (Gross, 1979). Yet the ability to establish motive deeply influences legal decision-makers, such as jurors, and is often a driver of case theory for attorneys. Why?

We put forward an adaptationist framework explaining one reason why people are preoccupied with knowing the motive behind an offender’s criminal behavior. The article begins by briefly considering the limited legal relevance of criminal motive, and by providing an overview of the psychological literature considering the impact of motive in this context. Then, the article shifts focus to theorize how an adaptationist framework could further contribute to the broader literature, arguing that people’s preoccupation with motive may be explained, in part, by the desire for information to assess perceived welfare trade-off ratios. We explain that motive matters because it provides information about the offender’s social value to others and the likelihood that the offender will impose costs on us in the future.

## 2. Motive: Legal Definition and Use in U.S. Criminal Law

Most introductions to criminal law in the U.S. establish actus reus (the guilty act) and mens rea (the guilty mind) as the key elements for proving guilt (e.g., Hessick, 2006). Actus reus refers to the physical actions, or conduct, that must occur to constitute the crime (LaFave & Ohlin, 2023). Mens rea is the internal element, reflecting the prerequisite state of mind or intentionality that must be established (LaFave & Ohlin, 2023). Notably, motive and intent are related (e.g., Minissale & Bergman Blix, 2025) in that they both require an examination of the internal workings of the defendant’s mind. However, they are distinct. Motive involves the underlying *reason* for a criminal act, whereas intent examines the *purposefulness* of the defendant’s behavior (Chiu, 2005). For example, imagine the defendant is on trial for stealing a wallet. The prosecutor must prove that the defendant both stole the wallet (actus reus) and intended to permanently deprive the owner of the wallet (mens rea). If the prosecution can demonstrate beyond a reasonable doubt to the legal decision-maker that the defendant committed the act with criminal intent, then the defendant should be convicted of the crime. As detailed in this example, the defendant’s motive for stealing the wallet (e.g., to buy illicit drugs or lifesaving medicine) is largely irrelevant to the legal analysis. In the words of Hessick (2006, p. 94), “[a] bad motive does not establish liability, and a good motive, standing alone, is not a defense to most crimes.”

A deeper examination, though, reveals the nuanced ways motive considerations can impact legal decision-making when determining guilt (e.g., Steiker, 1999). In some contexts, motive is incorporated into the elements of a specific crime (Hessick, 2006). Motive can be inculpatory or incriminating when an otherwise lawful behavior is performed with a specific motive, often identified as an “illegal purpose.” For example, federal law prohibits giving money to a public official if the funds are for the purpose of influencing or rewarding the official for acting in their official capacity (18 U.S.C. § 201(c)(1)(A), 2024). In this case, it is the motive or reasoning behind the contribution that takes an otherwise lawful action with lawful intent and makes it criminal. Motive can also function in an exculpatory fashion, justifying what would otherwise be criminal actions (Hessick, 2006). For example, there are a series of justification defenses to criminal charges, including self-defense. To claim these defenses, a defendant must demonstrate an appropriate motive, described as the “purpose requirement.” Essentially, the defendant must establish that they acted with the purpose of avoiding a more serious harm.

Thus far, the focus of this discussion has been on motive’s limited role in determining criminal liability. Motive arguably plays a larger role at the sentencing phase, after guilt has been established. In this context, motive can be factored in to establish the relative blameworthiness of the defendant, compared to others who commit the same offense (Steiker, 1999). The U.S. Supreme Court has confirmed that when a judge is considering what sentence to impose, a “defendant’s motive for committing [an] offense is one important factor” (Wisconsin v. Mitchell, 1993, p. 485). At the sentencing stage, motive can function as both an aggravating and mitigating factor (Hessick, 2006). For example, motives including financial gain, promoting terrorism, or a wish to inflict pain or harm can be reasons to support the imposition of a more severe sentence. In the case of mitigating factors, there are fewer mitigating motives clearly articulated in law, as this is most likely captured in “catch-all” provisions and case-by-case reasoning (Hessick & Berman, 2016). However, the Model Penal Code has codified the defendant’s belief that there was a moral justification or extenuating circumstance for their act as a mitigating motive in the provision on capital punishment (American Law Institute Act of 1962, Model Penal Code § 210.6(4)(d), 1962). In other contexts, the motive of helping the defendant’s family may also be considered a mitigating motive (e.g., United States Sentencing Commission, 2024).

Returning to the role of motive in adjudicating guilt, criminal motive may arguably be more impactful when serving an informal, persuasive role. Although criminal motive might be limited in its doctrinal function, it can increase legal professionals’ certainty around their decisions (Minissale, 2024). Further, motive is cited as influential for establishing a strong theory of the case, persuasive to jurors and other legal decision-makers. A quick online search returns numerous criminal defense attorney webpages detailing the importance of motive for “creating a narrative for the jury” (Hartsfield Law, 2025) and describe it as having “a powerful role in shaping how jurors interpret the facts and assess a defendant’s guilt or innocence” (Northrup, 2025). This aligns with attorneys’ training and practice of developing a “case theory” that depicts a central narrative of the case, often for the benefit of the jury (e.g., Thomas, 2008). Such a practice is also consistent with empirical findings supporting the Story Model for Juror Decision Making, which theorizes that jurors use narratives to organize and interpret evidence (Pennington & Hastie, 1992). Importantly, narratives presented in this research included information about the defendant’s motive. Altogether, this evidence suggests that attorneys believe motive plays a crucial role in legal decision-making, even when it is not an essential element of the crime. We next consider whether the psychological literature also supports this assertion.

## 3. A Brief Overview of the Psychology of Motive

Despite its general irrelevance for establishing criminal liability, information about an offender’s motive clearly affects the way we think about, judge, and behave towards the offender. Yet psychological research on this topic has been limited, and when it is studied the focus has primarily been on related concepts of blame and judgments of “character.” In order to understand *what* drives us to care about motive, it may be helpful to consider *how* exactly motive relates to our judgments of a respective action.

In an attempt to uncover this psychological process, Alicke (1992) considers one mechanism through which motive seems to influence blame. Namely, he theorizes that individuals with “bad” motives (versus “good” motives) are perceived as more *causally responsible* for an accidental outcome and more blameworthy. In the context of a car accident due to speeding, Alicke (1992) demonstrated that while the *reason* someone is speeding (to hide an anniversary gift or cocaine) is logically irrelevant to their causal impact on the accident that resulted, participants found the person with a bad motive as the primary cause of the accident more frequently and viewed that person as more blameworthy.

Using similar paradigms, Nadler and McDonell (2012) also found that individuals with bad motives were considered more causally responsible and blameworthy for the unintentional death of two individuals. Additionally, having “bad” moral character appeared to increase participants’ ratings of the actor’s negative intent and causal responsibility. The researchers concluded that the psychological process of blame seems to latch on to any potential indicator of character, whether that be through motive or other background information, which directs us towards not simply blaming the individual more, but biasing legally relevant components (i.e., causation or intent) as well (Nadler & McDonell, 2012). It is worth noting that both Alicke (1992) and Nadler and McDonell (2012) focused their experiments on situations in which both intent and causation were unclear (e.g., accidents and unintentional death), which is uncharacteristic of many criminal charges. Thus, although informative regarding the psychology of blame, it may suggest a potential limitation of this research when generalizing across crimes.

Further, although this work helps to demonstrate that motive does indeed play an important role in our judgments of acts, more mechanistic investigations are needed to better develop punishment theory. Again, the literature on blame may be instructive. For example, in their Path Model of Blame, Malle et al. (2014) consider motive (referred to as “reasons”) as fundamental in judgments of blame. Using a more general theoretical framework of social regulation, the researchers argue that the act of blaming assures that the behaviors of an individual align with the norms of the community. In their theoretical model, once a behavior is determined to have been intentional, one subsequently considers the agent’s reasons for committing an act. Malle et al. (2014) posit that reasons are essential for moral evaluations given that they help to reveal the feelings and attitudes of actors. Further, they provide insight into how to control the agent and anticipate future action.

Taking a similar approach, Nadler and McDonell’s (2012) theory of motive combines the social role of blame as well as the psychological phenomenon known as “motivated reasoning.” First, in reference to the work of Emile Durkheim and his theory of punishment, Nadler and McDonell (2012) note that there is a natural inclination to punish bad individuals to uphold the moral values of the society in which one lives (Durkheim, 1984). With this social imperative as a basis, the researchers describe how the process of determining blame is shaped by the psychological phenomenon known as motivated reasoning, a cognitive process that involves coming to a conclusion and subsequently construing the evidence in a biased manner such that it fits your preferred conclusion. Translating to a legal context, Nadler and McDonell (2012) argue that, through a process they call “motivated inculpation,” any potential indicators of bad moral character, such as motive (whether legally relevant or not), encourage a psychological biasing of legally relevant phenomena. That is to say, there is a subconscious attempt to validate our moral intuitions about the actor and hold legally responsible those whom we see as “bad.” Under this view, motive is important because it paints a picture of the actor’s moral standing, which helps determine whether they are worthy of punishment.

Why, though, is motive uniquely powerful information for determining the response an offender “deserves”? It may be of both practical and theoretical significance to push beyond the conclusion that we want to punish bad people because they are bad. What is needed is a framework that can explain the conditions under which we would predict motive to matter, as well as why the mind prioritizes information about prospective behavior (the offender’s future social value to the perceiver and broader community) rather than retrospective behavior (the committed act). We propose that an adaptationist framework can shed light on these important questions.

## 4. Applying the Adaptationist Framework to Criminal Law

An evolutionary psychological perspective offers a new lens for considering the importance of motive. This metatheoretical framework has been fruitfully applied to understanding a wide range of human psychology and behavior—from mate preferences to health behaviors, social influence, and financial decision-making (e.g., Neuberg et al., 2010)—including in the legal realm (e.g., Sznycer & Patrick, 2020; Williams et al., 2019). At its core, an evolutionary psychological approach begins with the assumption that behavior is functional—the ultimate function being reproductive fitness (successful perpetuation of one’s genes into subsequent generations; Neuberg et al., 2010). Through the process of natural and sexual selection, adaptations have evolved to address recurrent challenges to reproductive fitness. Just as physical features have evolved (e.g., eyelashes to protect the eye), *psychological* mechanisms have also evolved to facilitate survival and the achievement of reproductive goals.

The time scale over which human evolution has occurred is extremely long, spanning millions of years. This is an important tenet of evolutionary theory, because behaviors (and the psychological mechanisms that produce them) are in theory functional to the extent that they increased reproductive fitness *in the past*. Such a distinction is critical for two reasons.

First, it recognizes that the conditions under which ancestral humans evolved are very different from contemporary environments. The human mind evolved to enable ancestral humans to adaptively navigate within small-scale groups of between 25 and 200 individuals (Dunbar, 1993; Kelly, 1995; Petersen, 2015), not the massive anonymous societies we experience today. Importantly, this suggests that our psychology attends to factors that were adaptive under ancestral conditions, and not necessarily the same cues that we would predict would be relevant in a modern context (e.g., Petersen, 2013, 2015).

Second, and relatedly, it also recognizes that what was adaptive behavior in ancestral environments may nevertheless be maladaptive in contemporary environments. As a simple example, consider the consumption of sugary, fatty foods. In a relatively resource-poor ancestral environment, a preference for calorie-rich foods would have increased reproductive fitness. In today’s resource-rich environment, this same evolved preference can lead to overconsumption and negative health outcomes (Neuberg et al., 2010). As both these points suggest, a mismatch exists between our ancestral and modern environments, which may lead properly functioning evolved mechanisms to nevertheless produce maladaptive outputs (Li et al., 2018). In essence, due to the slow speed of evolution but rapid changes to our environment, our modern minds are still playing catch up.

In sum, an evolutionary perspective posits that the human mind possesses psychological adaptations that translate specific social and environmental inputs into outputs that were fitness-enhancing under ancestral circumstances (Tooby & Cosmides, 1990). One consequence is that our modern judgments may disregard modernly rational factors in favor of factors that were ancestrally relevant (Petersen, 2015). On the surface, this may seem to indicate an irrationality or dysfunctionality of our psychology given the present context. However, it may point instead to a deep rationality—a psychology evolved to operate in the context of small-scale ancestral life.

## 5. The Adaptive Challenge of Exploitation

One significant recurrent adaptive challenge faced by ancestral humans was exploitation by other people (i.e., costs imposed on the self for another’s own benefit; Petersen et al., 2010). Humans are a social species, and cooperation with others is critical to achieving our goals. However, organisms are also evolved to enhance their own self-interest. Cooperation therefore comes at the risk of social partners cheating, free-riding, and inflicting other costs. Faced with this reality, a critical adaptive problem is preventing exploitation. And indeed, evidence suggests that humans have evolved mechanisms that aid in the detection of cheating (Cosmides & Tooby, 1992), defense against freeriders, and the activation of moral outrage and punitive sentiments in response to exploitation (Petersen et al., 2010). Furthermore, the mind extends this desire for punishment beyond our own perpetrators to those who exploit others, because—absent evidence to the contrary—the mind infers that exploitation of a third party predicts later mistreatment of the self (Delton & Krasnow, 2017; Krasnow et al., 2016).

Faced with an offender, then, the mind should calculate the benefits of punishment over the potential costs (e.g., loss of a future cooperation partner, retaliation by the offender or their associates, etc.). Germane to this calculation is an assessment of the offender’s *association value*—the estimated lifetime value of maintaining a relationship with the offender from the self’s point of view (Tooby & Cosmides, 1996). Determining association value involves utilizing available cues in the environment to appraise the costs of losing the offender as a future social partner. This could involve assessing past behavior to infer the likelihood of future behavior, including assessing the likelihood that the offender will impose future costs. When the association value of an offender is high, people are more likely to experience reparative motivations that serve to preserve the relationship; when association value is low, punitive motivations are triggered (including, in extreme cases, expulsion or execution) (Petersen et al., 2010). Thus, it is the perception of an offender’s likely future behavior, rather than the seriousness of their act, that determines punishment proclivity (e.g., Petersen, 2007). Additionally, this explains why the same crime might inspire very different reactions depending on features of the perpetrator and the individual decision-maker, and it suggests that people should agree on appropriate sanctions to the extent that there is agreement on perceived association value of the offender (Petersen, 2013).

## 6. Welfare Trade-Off Ratios Guide Perception

How, though, does the mind determine one’s association value? The cues that feed into a calculation of association value are myriad (e.g., kinship, group membership, formidability, attractiveness, etc.), but one summary variable is particularly critical: The perception of a target’s welfare trade-off ratio (WTR) towards the self.

When making a choice that impacts another, an individual could take into account the extent to which that individual is willing to trade-off their own welfare against another’s welfare (e.g., Delton & Robertson, 2016; Sell et al., 2017; Sznycer et al., 2019; Tooby et al., 2008). This WTR factors in the other’s welfare to varying degrees that could be described as points along a continuum, including not at all, moderately, strongly, or even altruistically to their own detriment (Petersen et al., 2010). As a concrete example, researchers have measured WTRs by asking participants to make a series of financial choices. For example, do you get $5 or does your friend get $10? Do you get $10 or does your friend get $10? Do you get $15 or does your friend get $10? The participants’ responses can then be evaluated for ‘switch points,’ wherein they start favoring themselves over their friend (Delton & Robertson, 2016).

In decisions impacting others, we assess the WTR between ourselves and the person influenced by the act to determine whether we are placing sufficient weight on their welfare. For example, being willing to give up $15 so their friend could have $10 would suggest a greater WTR than being willing to give up $5. Similarly, when others act towards us, we evaluate the extent to which they are prioritizing our welfare in relation to their own. WTRs are not fixed, but instead update in response to changing information about people and relationships. The mind’s computation of WTRs occurs quickly, automatically, and without conscious effort (Del Ponte et al., 2021; Delton & Robertson, 2016).

Possessing a greater WTR towards another individual or group predicts more cooperative behavior and agreeableness (Delton & Robertson, 2016). On the other hand, there are severe potential fitness costs to interacting with people who do not care about our welfare (Petersen et al., 2010), as it functions as a strong predictive cue of exploitation. In other words, our inferences about future behavior are informed by WTRs. The mind should therefore be highly attuned to gauging the WTR that others express towards you (and those you value) by their actions. As Petersen et al. (2010) note, this suggests that we should be interested in behaviors that reveal WTRs and we should interpret and remember behaviors in terms of the WTRs they reveal.

In any circumstance where an action affects the welfare of an actor and a target, that action provides information about the WTR held by the actor towards the target, and, by extrapolation, the WTR the actor potentially holds towards an observer (Krasnow et al., 2016). Indeed, exploitation itself can be defined in relation to WTR, as by its nature it indicates a WTR that is low or negative towards an individual or group (Petersen et al., 2010).

In Delton and Robertson (2012), the researchers explored how perceived WTRs guide our inferences and willingness to cooperate with others. They hypothesized that people who were more willing to sacrifice their personal welfare would be seen by others as possessing a higher WTR towards the group, thus making them more desirable prospective social partners. For example, the researchers examined whether wading into shark-infested shallows to harvest crabs (a larger cost to the self) would be viewed more positively than cracking and breaking a watch while picking strawberries (a smaller cost to the self), despite resulting in equivalent contributions to the group’s shared resources. This was indeed the case. These findings suggest that the reverse should also be true: Individuals who promote, rather than sacrifice, their own personal welfare to the detriment of others should be inferred to have a lower WTR towards the group. In essence, the decisions made by other people contain information about how much value they assign to our (and our group’s) welfare (Quillien et al., 2023). Perceivers extract this information and use the imputed WTRs to modify their own future behaviors towards that individual accordingly.

Inferring how much weight someone puts on one’s own welfare is a complex process rife with uncertainty. But given the evolutionary importance of valuation estimation, the mind should have evolved machinery that makes these inferences in an efficient and rational way (Quillien et al., 2023). In the context of evaluating an offender’s actions, one cue that should significantly shape inferences of WTRs is the perceived motive of the offender.

## 7. Motive and WTRs

At its core, motive is about reason—what were the offender’s *reasons* behind the offensive act? Did you borrow my favorite sweater to wipe your mouth or to staunch the bleeding of your child’s wound (Quillien et al., 2023; Sell et al., 2017)? In both cases, the outcome (or, “crime”) is the same, but these two actions have very different implications for inferring WTRs. If you used my sweater to wipe your mouth, this suggests that you possess a low WTR towards me and would not hesitate to impose costs upon me for your own benefit (however small), now or in the future. From this motive one can infer information about the likelihood of a future offense. The same inference is unlikely if your motivation was to save your child’s life.

Our minds intuitively conduct a cost–benefit analysis sensitive to valuations, such that a formerly unacceptable cost (a ruined sweater) might become acceptable if you detect a significant benefit to the actor (saving a child) (Petersen et al., 2010). The same logic applies to using behavior directed against third parties to gauge WTRs towards the self or your group. If Anna uses Bridget’s sweater as a napkin, an observer can impute not only Anna’s WTR towards Bridget but potentially Anna’s WTR towards the observer and their group, to the extent that WTRs towards third parties predict dispositions towards the self and others (Krasnow et al., 2016; Petersen et al., 2010). Motive therefore provides critical insight into the decision-making processes of the perpetrator—their calculation of the magnitude of the costs imposed in proportion to the benefits received, given the specific target of the action (Sell, 2005).

A prediction, then, is that motive matters to the extent that it provides information about the relative costs and benefits experienced by the parties involved. Circumstances in which one party imposes large costs on another to gain a much smaller benefit for themselves should be seen as especially problematic and worthy of punishment (Petersen et al., 2010). Thus, an offender who steals $20 to buy illicit drugs may generate more outrage and punishment than an offender who steals $20 dollars to purchase lifesaving medicine (Rossi et al., 1985). Although the magnitude of the cost is the same, the benefits generated for the perpetrator in proportion to the cost inflicted are different, leading to divergent perceptions of the perpetrator’s WTRs. Further, the motives imply a different likelihood of committing the same crime in the future.

The motive behind an exploitative act may also help observers infer whether the offender possesses a general exploitative disposition or whether the act was specific to the relationship between the offender and victim (Panchanathan & Boyd, 2003). In other words, is it only the victim towards whom the offender has a low WTR, or is it towards all members of a particular group (which may include oneself)? This leads to the prediction that motives indicating a special relationship between the offender and victim may generate less punitiveness than motives indicating a more generalized criminal disposition (Petersen et al., 2010). For example, an offender who kills his boss for failing to give him a raise is likely to generate less outrage than an offender who kills the fourth man who walks by him on the street (see, e.g., Rossi et al., 1974).

In sum, an adaptationist framework suggests that evolution has designed mechanisms in the human mind that infer the welfare valuation between individuals, and the mind uses this information to reach decisions about how oneself should treat others in turn. Motive, in particular, provides critical information about how much an offender values others (including oneself)—with implications for the benefits and costs of future interaction with the offender—and is therefore given substantial weight in a punisher’s decision-making process.

## 8. Discussion

Here, we have considered the question of why an offender’s motive plays a substantial role in people’s responses to a harmful act. Although limited in number, previous articles exploring this question have suggested that motive is important for shaping the causal inferences that are made about offenders’ actions: When offenders’ motives are perceived as “bad,” those offenders are seen as having played a more causal role in the negative outcome produced by their actions and they are considered more blameworthy. This phenomenon occurs because offenders with bad motives are viewed as possessing bad character, and we are intrinsically motivated to punish such actors for their moral failings.

We then introduced an alternative perspective for considering the importance of motive: An adaptationist framework, which focuses on the potential fitness consequences of cognition and behavior. We proposed that evolved psychological mechanisms for hindering exploitation may help explain our attunement and reactions to the inferred motives of criminal offenders. In particular, we posited that motive matters because it feeds into the calculation of an offender’s association value, specifically by informing our perceptions of an offender’s welfare trade-off ratio (WTR) towards a victim or group, and, by extension, oneself and one’s group. The primary novel contribution generated via this framework is that motive is highly informative for valuation, and *valuation* is what primarily drives our willingness to punish–not, e.g., severity of offense, perceived fault, or desire for retribution (see, e.g., Petersen et al., 2012).

The significant implications of this deduction are fourfold. First, it suggests that we can find perpetrators with divergent motives similarly responsible for an offensive act, yet reach very different conclusions about the offenders’ WTRs. For example, perhaps both P’s and Q’s actions cause a sweater to be ruined, but their different motives (wiping her mouth versus staunching a bleeding wound, respectively) will lead us to perceive a low WTR from P, but not from Q. This, in turn, should drive our desire to punish P and forgive Q.

Second, as demonstrated by the sweater example above, the perceived ratio of costs to benefits to the parties matters to observers. If an action produces a great benefit for the perpetrator (e.g., saving a loved one’s life), the more acceptable the motive is perceived to be, even holding costs constant (a ruined sweater). Said another way, we are more forgiving of motives that produce greater benefits for offenders because such motives do not imply a low WTR to the same degree as motives that produce smaller benefits (while inflicting the same amount of costs).

Third, the emphasis on valuation implies that if the offender’s future behavior is anticipated to be benefit-enhancing for the decision-maker or the decision-maker’s associates, even a “bad” motive will result in less punishment as compared to an offender who is seen as posing future costs. Implicit in this is the recognition that the decision-maker is inferring the offender’s future behavior toward the decision-maker, based on their valuation. Thus, as long as the perceived WTR between offender and self remains high, our inclination will be reparative rather than punitive (e.g., Petersen et al., 2012).

Fourth, and relatedly, this suggests that if an offender’s association value is high (say, for example, because they are your genetic relative), the offender’s motive will be less influential on your decision to punish than if the offender’s association value is low. Put plainly, the relationship between a perpetrator and a decision-maker matters for assessing the significance of their motive. The legal system implicitly acknowledges this reality by excusing judges and jurors with conflicts of interest from serving as decision-makers on those cases. However, the full nature and extent of the role of association value in decisions of guilt or punishment–and how this may intersect with motive–has not yet been tested empirically. Ultimately, empirical studies are needed to evaluate all four implications outlined here, and to test between alternative accounts of the importance of motive. This area of inquiry is ripe for additional research.

The idea proposed here–that motive informs WTRs, that WTRs shape valuation, and that valuation drives determinations of guilt and punishment–is not wholly inconsistent with current practices in the criminal justice system. Although most crimes do not require a demonstration of motive to prove guilt, there are crimes for which motive is deemed important enough to become an element of the crime (e.g., so called “hate crimes”; Hessick, 2006). Additionally, there are situations such as self-defense where motive can serve an exculpatory function, justifying what would otherwise be a criminal action. In these exceptional cases, one could argue that the valuation analysis is seemingly codified into the laws.

Here, we have posited that jurors and other legal decision-makers unconsciously incorporate motive information into their computation of WTRs, and that these appraisals might ultimately influence their guilt determinations. Yet it is also worth acknowledging that motive’s largest role in criminal law is at the sentencing phase, when the severity of punishment is determined (Steiker, 1999). At sentencing, decision-makers often have discretion to consider motive as either an aggravating or mitigating factor (Hessick, 2006). It is at this phase that the valuation associated with WTRs and perceptions of future costs may be more apparent, affecting punishment decisions. As sentencing is most often carried out by a judge (Sporer & Goodman-Delahunty, 2009), this brings up interesting questions about whether judges would make similar judgments as the general public. On the one hand, judges’ formal training and experience might inoculate them against the influence of WTR-based valuation. On the other hand, judges possess the same evolved psychological mechanisms as laypeople, and research suggests that expertise is not always a panacea for bias (e.g., Guthrie et al., 2001). Empirical studies are needed to test between these possibilities.

There are also interesting questions about the implications of motive for plea bargaining in light of WTRs. Around 90% of federal criminal cases involve the defendant pleading guilty (Gramlich, 2023). Although the exact number is difficult to ascertain based on court provided data, many guilty pleas are the result of plea bargaining, a process in which prosecutors have considerable discretion in determining the charges and sentence (Bibas, 2012). To what extent does an offender’s purported motive shape prosecutors’ perceptions of WTRs, potentially influencing plea decisions? Conceivably, “bad” motives could impact prosecutors’ discretion regarding the severity of the charges and recommended punishment. Indeed, the literature from practicing attorneys suggests that both prosecutors and defense attorneys are attuned to the implications of motive for crafting a persuasive narrative (e.g., Thomas, 2008). Thus, prosecutors might perceive the existence of a “bad” motive as one tool in their toolbox for securing convictions, and the presence of a “good” motive–or the absence of a motive entirely–as one reason to avoid the uncertainties of trial.

In this article we have largely focused on crimes that involve a clear imposition of harm towards an identifiable victim. However, some crimes do not require a particular result to occur. For example, it is illegal to drive drunk even if you are flawlessly operating the vehicle. We propose that these and other ‘victimless’ crimes might also convey WTR information to observers. For example, one’s willingness to risk potential harm to others by driving drunk conveys how one weighs the welfare of others compared to one’s own welfare.

Although we have focused herein on motive, additional legally relevant variables are also likely to influence one’s WTR calculus. For example, information about an offender’s past offenses has significant implications for estimating the likelihood of future cost infliction. Indeed, the law recognizes the highly prejudicial nature of this information, and federal evidentiary rules prohibit its admission when determining guilt except under narrowly defined circumstances (Federal Rules of Evidence, Rule 404, n.d.). Interestingly, this is a space that intersects with motive. Under Rule 404(b)(1), an offender’s specific previous acts of misconduct are generally inadmissible if the purpose of offering them is solely to establish a criminal disposition or bad character. However, this information can be admissible if deemed ‘independently relevant’; one such example of relevance is if the acts of misconduct establish motive in the present case (Rule 404(b)(2)). And, notably, these rules of evidence typically do not apply at the sentencing phase, where motive and character information are often relied upon as relevant data points.

Finally, the potential practical implications of this research are numerous. Insights into how people weigh an offender’s future association value could help policymakers and courts craft policies and laws that better account for the influence of specific cues. For example, depending on the goals of the decision-maker, this could shape policies concerning the admission of certain types of evidence (e.g., motive information) at both the guilt and sentencing phase. This area of research could also inform legal practice, potentially influencing attorney arguments and strategies. A defense attorney advocating for their client may want to take into consideration decision-makers’ WTR calculus when building their case narrative. By highlighting motives that provide critical insight into the defendant’s calculation of the magnitude of the costs imposed in proportion to the benefits received, the attorney could sway the WTR calculation in favor of their client. The same would be true for prosecutors trying to persuade the decision-maker to their side. Lastly, at a more general level, research through this lens would deepen our understanding of the psychology of punishment, furthering ongoing discussions of the philosophy of punishment.

## 9. Conclusions

Motive occupies an interesting status in the American criminal justice system. It is usually—though not always—irrelevant to the legal analysis of establishing guilt. Yet its role in the human psyche is profound. As perceivers, we intuitively seek information about why people behave the way that they do, and we use this information to inform our perceptions of responsibility and punishment. Thus, the psychological role motive plays is somewhat incongruous with its role in the legal process associated with determining guilt.

Here, we introduced a novel approach for considering the significance of motive: An adaptationist framework that considers how our minds have been shaped across evolutionary time by natural and sexual selection. We proposed that our modern preoccupation with motive may be connected to psychological mechanisms that evolved to address the recurrent fitness challenge of avoiding exploitation. In particular, we described how the mind weights an offender’s future association value in determining punishment, and that these calculations are strongly informed by cues of how much an offender values the decision-maker’s welfare. Motive serves as one such critical cue. Our contemporary concern with motive may therefore be evidence of a deeply rational evolved psychology, rather than, for example, a dysfunctional inability to ignore ‘legally irrelevant’ information.

Ultimately, applying this evolutionary lens provides a strong framework for developing novel, testable hypotheses and thus building a greater understanding of motive’s psychological relevance and influence in legal decision-making.

## Data Availability

No new data were created or analyzed in this study. Data sharing is not applicable to this article.
